# Transcriptional Regulation of Energy Metabolism in Cancer Cells

**DOI:** 10.3390/cells8101225

**Published:** 2019-10-09

**Authors:** Sara Rodríguez-Enríquez, Álvaro Marín-Hernández, Juan Carlos Gallardo-Pérez, Silvia Cecilia Pacheco-Velázquez, Javier Alejandro Belmont-Díaz, Diana Xochiquetzal Robledo-Cadena, Jorge Luis Vargas-Navarro, Norma Angélica Corona de la Peña, Emma Saavedra, Rafael Moreno-Sánchez

**Affiliations:** 1 Departamento de Bioquímica, Instituto Nacional de Cardiología, México 14080, Mexico; marinhernndez@yahoo.com.mx (Á.M.-H.); jcga_1999@yahoo.com.mx (J.C.G.-P.); suerte11@hotmail.com (S.C.P.-V.); belmont81@hotmail.com (J.A.B.-D.); xochiquetzal@ciencias.unam.mx (D.X.R.-C.); jorge.navarro6@hotmail.com (J.L.V.-N.); emma_saavedra@hotmail.com (E.S.); rafael.moreno@cardiologia.org.mx (R.M.-S.); 2 Unidad de Investigación Médica en Trombosis, Hemostasia y Aterogénesis, Hospital General Regional Carlos McGregor-Sánchez, México CP 03100, Mexico; norma_acp@hotmail.com

**Keywords:** cancer transcriptional regulators, energy metabolism regulation, glycolysis, oncogenes, oxidative phosphorylation, transcriptional factors

## Abstract

Cancer development, growth, and metastasis are highly regulated by several transcription regulators (TRs), namely transcription factors, oncogenes, tumor-suppressor genes, and protein kinases. Although TR roles in these events have been well characterized, their functions in regulating other important cancer cell processes, such as metabolism, have not been systematically examined. In this review, we describe, analyze, and strive to reconstruct the regulatory networks of several TRs acting in the energy metabolism pathways, glycolysis (and its main branching reactions), and oxidative phosphorylation of nonmetastatic and metastatic cancer cells. Moreover, we propose which possible gene targets might allow these TRs to facilitate the modulation of each energy metabolism pathway, depending on the tumor microenvironment.

## 1. Introduction

All stages of cancer progression, including initial formation, growth, and metastasis (i.e., epithelial to mesenchymal transition, adhesion protein expression, migration, invasiveness, colonization, and angiogenesis), are regulated and coordinated by several transcriptional regulators (TRs), including transcriptional factors (TFs), transcriptional coactivators, oncogenes, tumor-suppressor genes, and protein kinases [1,2,3]. The term “transcription factor” describes any protein able to bind to DNA in a sequence able to regulate transcription or alter gene expression. “Transcriptional coactivator” describes any protein or protein complex that binds to a transcription factor to increase the transcription rate of a gene or set of genes. “Oncogene” describes a gene whose protein product carries the ability to induce cancer and confer at least one aspect of the transformed phenotype on a cell. “Tumor-suppressor gene” describes a gene whose encoded protein protects a cell from one step on the path to cancer. Cancer progresses when oncogene function results from the activation of proto-oncogenes or when tumor-suppressor genes are inactivated.

The roles of transcriptional regulators (TRs) in cancer development and metastasis have been well documented and analyzed [3,4]. Besides their allocated canonical roles (Table 1), TRs may also be involved in the regulation of other important tumor processes that have not been widely examined. One of these processes that becomes relevant for cellular homeostasis and essential for intense cell activities such as accelerated proliferation (cancer), mechanical contraction (heart), and secretion (brain) is adenosine triphosphate (ATP) production in the cytosol (glycolysis) and mitochondria (oxidative phosphorylation, OxPhos). Moreover, an ATP supply is also required for other high-energy-demanding processes such as the biosynthesis of macromolecules (proteins, nucleic acid, glycogen, and lipids), ion homeostasis, apoptosis, cell death evasion, and metastasis onset.

Thus, the present review gathers and analyzes information indicating that some TFs, oncogenes, tumor-suppressor genes, and other proteins (kinases and plasma membrane receptors) associated with cancer progression and metastasis have canonical roles that overlap with the regulation of the cellular ATP supply by modulating OxPhos and glycolytic fluxes in cancer cells depending on microenvironmental conditions. The identification of TRs of energy metabolism pathways in cancer cells may help to improve our understanding of the control and regulation mechanisms specifically working in cancer cells (compared to healthy cells), thus leading to potential novel metabolic therapeutic targets.

## 2. Transcriptional Regulators of Glycolytic Flux in Cancer Cells

### 2.1. Transcriptional Factors

#### 2.1.1. Hypoxia-Inducible Factor-1 (HIF-1)

HIF-1 is a heterodimeric protein constituted of two subunits: the low oxygen-induced isoform HIF-1α and the constitutive isoform HIF-1β. The HIF-1 dimer is able to interact with the promoter regions of specific nuclear genes, modulating their transcription. The canonical target cell functions in which it is involved are listed in Table 1. *HIF-1A* gene transcription is constitutive, but active degradation of its protein maintains it as low under normoxia in normal cells. HIF-1α protein hydroxylation by prolyl hydroxylases (PHs) triggers its ubiquitination and further proteasomal degradation [5]. PHs require 2-oxoglutarate (2-OG), O_2_, Fe^2+^, and ascorbate for activity. Due to their high *Km* values for O_2_ (>200 μM), PH activity is highly modulated by intracellular [O_2_] [6]. The physiological [O_2_] range is 50–100 μM in aerobic tissues and organs [7,8], and hence, under hypoxia ([O_2_] < 10 μM), PH activity becomes suppressed, allowing for HIF-1α stabilization in noncancer cells and tissues.

In contrast, HIF-1α can be stabilized under both normoxia and hypoxia in cancer cells. Thus, high HIF-1α protein levels are usually detected in metastatic cancers, whereas comparatively much lower HIF-1α protein is detected in both benign cancers and noncancer cells [9,10]. Under normoxia, glycolytic flux increases in cancer cells, leading to elevated cytosolic pyruvate and lactate levels, which are PH competitive inhibitors versus 2-OG [11]: other PH inhibitors such as succinate and fumarate may also be elevated in cancer cells [12,13]. In addition, the heightened reactive oxygen species (ROS) levels found in malignant tumors [14] can also inhibit PH activity [15] because catalytic-site cysteine residue becomes oxidized. Furthermore, to contend with ROS overproduction, high intracellular ascorbate, cysteine, and glutathione are required. In consequence, PH activity is limited by substrate- (ascorbate) and catalytic-site cysteine in its reduced form (-SH) versus its oxidized form (-SO_x_). This PH inactivation blocks HIF-1α degradation in cancer cells [5,6].

**Figure 1:**
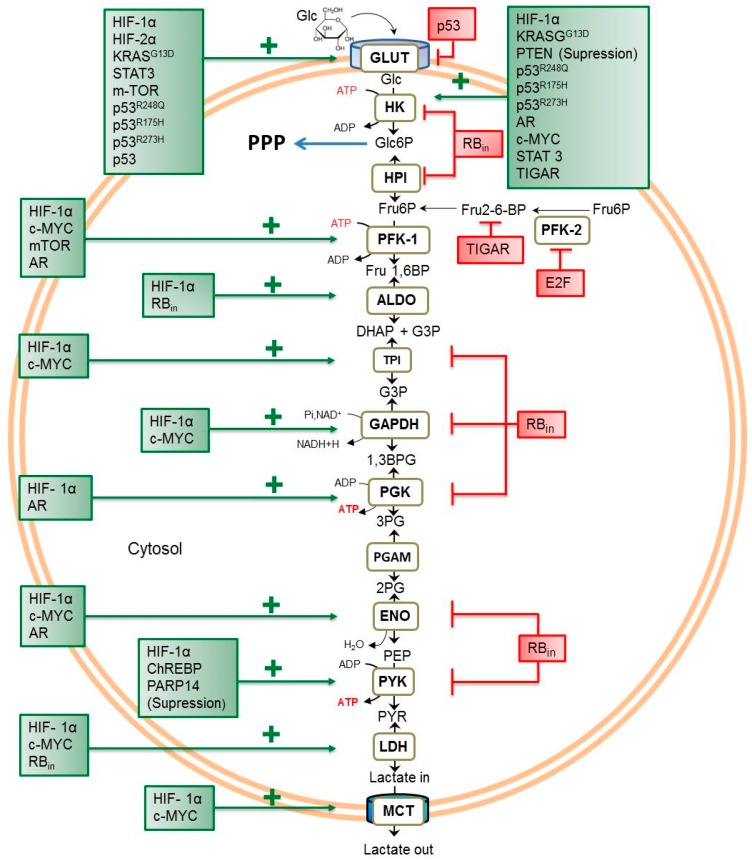
Transcription regulators (TRs) that modulate glycolytic metabolism in cancer cells. Red boxes and lines represent TRs with inhibitory effects, and green boxes and arrows represent TRs with activation effects. Abbreviations: 1,3BPG, 1,3-bisphosphoglycerate; 2PG, 2-phosphoglycerate; 3PG, 3-phosphoglycerate; ALDO, aldolase; DHAP, dihydroxyacetone phosphate; ENO, enolase; Fru1,6BP, fructose1,6-bisphosphate; Fru6P, fructose6-phosphate; G3P, glyceraldehyde-3-phosphate; GAPDH, glyceraldehyde-3-phosphate dehydrogenase; Glc, glucose; Glc6P, glucose6-phosphate; GLUT, glucose transporter; HK, hexokinase; HPI, hexose phosphate isomerase; LDH, lactate dehydrogenase; MCT, monocarboxylate transporter; PEP, phosphoenol pyruvate; PFK1, phosphofructokinase type 1; PGAM, phosphoglycerate mutase; PGK, phosphoglycerate kinase; PPP, pentose phosphate pathway; PYK, pyruvate kinase; PYR, pyruvate; TPI, triosephosphate isomerase.

Most of the genes encoding glycolytic enzymes and transporters are targets of HIF-1α in normal and cancer cells (Table 2, Figure 1), except for those coding for hexose-phosphate isomerase (HPI) and monocarboxylate transporters (MCT) (*GPI* and *SLC16* or *SLC5* genes, respectively). Therefore, the higher levels of HIF-1α in cancer cells regardless of normoxia or hypoxia correlate with increased levels of glycolytic proteins. For instance, under hypoxia, the much greater HIF-1α versus normoxia content correlates with higher glycolysis rates as well as extracellular acidosis derived from the enhanced lactate plus H^+^ production and ejection [39,40] (Table 2). Similarly, it has been reported that hypoxia also increases glycogen synthesis mediated by enhanced HIF-1α stabilization in cancer (mouse hepatoma HePaC1; breast MCF-7 and MDA-MB231; colon LS174 and BE; and kidney RCCA) and noncancer (lung CCL39; mouse embryonic fibroblasts (MEFs); mouse skeletal myoblast C2C12; myotubes; mouse hepatocytes) cells: HIF-1a regulation of glycogen metabolism in cancer cells under normoxia has not been explored. Indeed, transcription of the genes coding for phosphoglucomutase (PGM) and glycogen synthase is also regulated by HIF-1α [41,42,43]. In consequence, increased glycogen synthesis and its specific metabolite pool levels are observed in both cancer and noncancer cells under hypoxia and with a sufficient external glucose supply (Table 2).

In addition to HIF-1α, other proteins involved in the hypoxia response are HIF-2α and HIF-3α. All three HIFs with an oxygen-sensing α-subunit require a common and stable β-subunit. Although hypoxia increases HIF-2α protein content [44,45], this TF does not affect the mRNA content of several glycolytic enzymes (PGK1, PGM-1, PYKM, LDH-A) in breast cancer (MDA-MB-231 and MDA-MB-468) and normal (epithelial) cell lines [44,45]. In HIF-1α-inactivated renal 786-0 carcinoma, HIF-2α increases the GLUT1 mRNA and protein contents by at least two times (Table 2) [45], an apparent compensation mechanism. However, this GLUT1 increment may be related to the activation of cellular pathways for cellular survival under stress conditions. Indeed, GLUT overexpression decreases hypoxia-induced apoptosis by downregulating the c-JNK-NH2-terminal kinase or inhibiting cytochrome c release through AKT pathway activation [44].

While HIF-1α and HIF-2α work as key regulators of the transcriptional response to hypoxia, much less is known about HIF-3α [46]. The *HIF3A* gene gives rise to multiple variants, which are expressed in different tissues at different developmental stages and are differentially regulated by hypoxia. Some HIF-3α variants may downregulate or completely inhibit HIF-1/2α actions by competing for the common HIF-β subunit [46]. Therefore, it seems possible that HIF-3α may act as a strong inhibitor of glycolysis. However, there is no information available on the effect of HIF-3 on cancer glycolysis.

#### 2.1.2. p53 Wild-Type and Mutant Isoforms

The homotetrameric tumor suppressor p53 protein, coded by the *TP53* gene, has 12 different isoforms (p53α, p53β, Δ40p53γ, Δ133p53α, Δ133p53β, Δ133p53γ, Δ160p53α, Δ160p53β, and Δ160p53γ), and p53α is the most abundant and well-studied [77]: p53 acts as a TF of several cellular processes associated with cancer suppression (Table 1). In tumors, p53 is found in both nonmutant and mutant (R175H, H179R, R181H, R249S, R273H, R248Q, and R280K) isoforms. Although both nonmutant and mutant p53 are found in malignant cancers, 80% of most malignant breast, colon, and ovary carcinomas show at least one mutation in the p53 protein [78]. In cancer cells, severe hypoxia (O_2_ = 0.02–0.1%) stabilizes p53 through stress-induced covalent modification. Phosphorylation (at serine 15) activates p53, inducing its nuclear accumulation [79] to activate transcription of its target genes (Table 1, Figure 1).

At the energy metabolism level, nonmutant/mutant p53 regulatory functions depend on oxygen availability [50]. Under normoxia (21% O_2_), the overexpression of the nonmutant p53 isoform in sarcoma Saos-2, cervix cancer HeLa cells, and immortalized MEFs correlates with a significant decrement in GLUTs (1, 3, and 4) and PGAM protein contents as well as in PGAM activity, but glycolytic flux remains unchanged (Figure 1, Table 2). In addition, mitochondrial protein contents (2OGDH, GA, and ND1) and OxPhos flux increase (Table 3) by at least three times. On the contrary, when oxygen availability decreases to 0.1–1%, nonmutant p53 overexpression induces increased glycolytic protein contents (GLUT1 and GLUT3), but HKII remains unchanged and glycolytic flux significantly decreases (Table 2). Apparently, a direct interaction between p53 and HIF-1α occurs, disabling the HIF-1α metabolic function through p53 sequestration. On the other hand, the OxPhos protein contents and flux decrease (Table 3), leading to an energy metabolism reprogramming in which glycolysis becomes the main ATP provider [50].

It has been documented that R175H, R248Q, and R273H mutations of p53 increase the glycolytic flux versus nonmutant p53 (Table 2). Higher glycolytic rates correlate with increased GLUT1, GLUT3, HKI, and HKII protein levels in mutated p53^R248Q^-containing cells under normoxic and hypoxic conditions (Table 2), thus also leading to an energy metabolism reprogramming by predominant glycolytic phenotype independent of oxygen availability [52].

TIGAR (TP53-induced glycolysis regulatory phosphatase) is a protein that is overexpressed in cancer cells that is transcriptionally regulated by nonmutant p53 and associated with drug resistance (Table 1). TIGAR protein expression is induced in cancer cells (a) by direct transcriptional induction by hypoxia, which involves p53 binding to the *TIGAR* gene promoter [123]; or (b) through activation of the AMPK/p53 signaling pathway under hypoxia or hypoglycemia [124]. Once TIGAR increases in the cytosol of hypoxic cells, it interacts with the mitochondrial bound HKII, increasing its activity (Table 2) [63]; however, in some studies glycolytic flux was not measured to assess the TIGAR role in regulating metabolic functions. Under normoxia, TIGAR downregulates glycolysis flux because it decreases the F2,6BP level, which is a potent allosteric activator of PFK-1 in cancer and noncancer cells (Figure 1, Table 2). As a consequence of TIGAR overexpression and glycolysis downregulation, PPP (pentoses-phosphate pathway) is strongly activated by G6P accumulation (Figure 1) [31], favoring cellular NADPH overproduction through the stimulated activity of Glc6PDH and 6PGDH in decreasing ROS levels. Therefore, cancer cells may become resistant to any insult associated with oxidative stress [125].

#### 2.1.3. PGC1α

PGC1 (peroxisome proliferator-activated receptor γ, coactivator 1) designates a family of transcriptional coactivators (PGC1α, PGC1β, and PGC1-related coactivator) that interact with TFs and nuclear receptors to exert their biological functions (Table 1) [18]. The most well-known and -studied member of the PGC1 family is PGC1α, although other isoforms such as PCG1α-b, PCG1α-c, NT-PGC1α, PGC1α2, PGC1α3, and PGC1α4 are also members of the PGC1a subfamily [126]. This TF is overexpressed in some cancers (prostate, breast) [18]. Hypoxia (1% O_2_/24 h) increases PGC1α and mitochondrial biogenesis in human hepatocarcinoma as a survival mechanism [127]; however, the effects of PGC1α on cancer glycolysis are unknown. In human muscle biopsies, PGC1α increases GLUT4 and HKII protein levels, which correlates with an increased activity of the PPP enzyme Glc6PDH, although the PPP net flux rate remains constant [128]. The principal PGC1α targets are those related to mitochondria biogenesis and OxPhos activation in cancer cells (see Section 3).

#### 2.1.4. NF-κB

NF-κB (nuclear factor kappa light chain-enhancer of activated B cells) is involved in the transcriptional regulation of several cellular processes associated with cancer progression (Table 1). NF-κB subunits p50, p52, RelA, RelB, and c-Rel contain recognition sites for promoters of target genes [129]. To be transcriptionally active, NF-κB requires only two subunits to form homo- or heterodimeric proteins [130]. Some breast, lung, and ovary cancers contain mutations in the RelA subunit [131,132]. RelA overexpressing MEFs show an accelerated extracellular glucose consumption and higher lactate and intracellular ATP production than do normal MEFs. Thus, it has been proposed that, similarly to HIF-1α, NF-κB also plays a pivotal role in reprogramming tumor glycolysis [133]. NF-κB is strongly induced by hypoxia (0.1% O_2_) through the activation of its regulatory proteins, the IkB and IκBα kinases (also named IKKs). The *GLUT3* gene contains response elements to NF-κB in its promoter. NF-κB activation is negatively regulated by mutant and nonmutant p53 because p53 blocks the complex (p65/IKKα/phosphorylated histone H3) formation needed for NF-κB transcriptional activity. In consequence, the loss in p53 function upregulates the NF-κB signaling pathway through IKK activation [51,133].

#### 2.1.5. TFAM (Transcription Factor A, Mitochondrial)

TFAM (transcription factor A, mitochondrial) is constituted by two isoforms, the large homodimer TFAM protein and the short Δ5TFAM protein, with the former being the most-studied isoform [134]. Although the principal function of TFAM is to stabilize mitochondrial DNA [135] and favor mitochondrial function (for details, see Section 3), other TFAM physiological roles have been recently unveiled (Table 1). For example, in breast MDA-MB-231, T47D, MCF-7, and MDA-MB-453 cancer cells, TFAM is involved in cell growth and metastasis progression [21]. In addition, a diminution of 30–70% in the content of TFAM correlates with a similar decrease in glycolytic flux (Table 2) in low metastatic lung A549 and H460 carcinomas [54]. The molecular mechanisms behind glycolysis arrest induced by TFAM have not been elucidated in cancer cells. However, in brain mouse tissue, moderate hypoxia (8% O_2_/24h) increases *TFAM* gene mRNA levels and mitochondrial biogenesis similarly to PGC1α (Section 3.1.3) as a survival mechanism [136].

#### 2.1.6. STAT3

STATs (signal transducer and activator of transcription) are a family of seven (STAT1–4, -5a, -5b, and -6) transcription factors involved in multiple cellular processes (Table 1), with STAT3 and STAT5 being the most highly overexpressed and activated in malignant cancers [55]. STAT3 has two isoforms (α and β), and STAT3α is the most-studied isoform involved in cancer progression [137]. After activation by cytokines, growth factors, and JAKs (Janus-kinases), the dimeric form of STAT3 binds to promoters of their gene targets for gene transcription activation [138]. In some metastatic and cancer stem cells (bladder, colon, lung, and breast carcinomas), phosphorylated STAT3 (pSTAT3) promotes cancer proliferation by upregulating antiapoptotic proteins (BCL-2, BCL-xl), pluripotency markers (OCT4), and proto-oncogenes (*MYC*) [22,23]. In liver carcinomas, pSTAT3 increases HKII mRNA and protein contents (Figure 1, Table 2): pSTAT3 also increases HIF-1α and GLUT1 mRNAs in HBV and HCV virus-related hepatocarcinoma and upregulates both glucose consumption and lactate production in HepG2 and Hep3B hepatocellular cancer cells (Table 2), most likely by increasing HIF-1α-mediated transcription of most glycolytic genes.

#### 2.1.7. FOXO-1

The FOXO (forkhead box) family is constituted by FOXO-1, FOXO-3a, FOXO-4, and FOXO-6 proteins [139]. The transcriptional regulation functions of canonical monomeric FOXO-1 are listed in Table 1. It has been demonstrated that FOXO-1 (i) decreases the mRNA levels encoding enolase (ENO) and pyruvate kinase (PYK) in mice livers [140] and that it (ii) is the target of AKT phosphorylation, blocking the transcription of glucose-6-phosphatase (Glc6Pase)- and phosphoenolpyruvate kinase (PEPCK)-encoding genes in hepatocytes [141]. Unfortunately, protein contents and pathway fluxes have not been assessed in parallel to rigorously establish whether FOXO-1 may regulate the glycolytic pathway. In cancer cells, the role of FOXO-1 in glycolysis has not yet been elucidated.

#### 2.1.8. E2F

The E2 family of transcription factors (E2F) is involved in the control of cell cycle progression. This family is constituted by activator (E2F1, E2F2, E2F3a, and EsF3b) and repressor (E2F4, E2F5, E2F6, E2F7, and E2F8) proteins. Both E2F1 and E2F3 are monomeric proteins [142]. Their actions are modulated by the RB protein, regulating cell proliferation and angiogenesis (Table 1). In metastatic cancers (nonsmall cell lung cancer; glioblastoma; pancreatic ductal carcinoma; and bladder, breast, ovarian, or prostate cancer), E2F1 and E2F3 are highly overexpressed [143]. In rhabdomyosarcoma cells, E2F1 downregulates fetal-type PFK2/F2,6BPase expression (Figure 1, Table 2), although PFK1 activity and the glycolysis rate have not been experimentally evaluated. It has also been documented in 3T3 mouse fibroblasts that E2F upregulates fetal-type PFK2/F2,6BPase during cell division [144], which may inhibit the glycolytic flux and stimulate gluconeogenesis, but the E2F role in cancer cells has not been analyzed.

#### 2.1.9. AR (Androgen Receptor)

Androgen receptor (*AR*) gene transactivation requires (i) androgen binding; (ii) translocation from the cytosol to nuclei; (iii) homodimer formation (two AR proteins and one androgen); and (iv) DNA binding [145]. In the human androgen-dependent prostate cancer cells LNCaP and LAPC4, AR activation with the synthetic androgen R1881 increases the HKII, PFK-platelet isoform, ENO, and PGK mRNA contents versus nontreated cells (Figure 1, Table 2), as well as the glycolytic rate, which is measured as the extracellular acidification rate (Table 2). Hypoxia increases the levels of the AR ligand peroxiredoxin 1, favoring AR activation in prostate (LNCaP and LAPC4) cancer. Thus, under hypoxia, perhaps AR contributes to global glycolysis activation at least in prostate cancer cells.

#### 2.1.10. ChREBP

The carbohydrate-response element-binding protein (ChREBPα and ChREBPβ) is a TF regulating several cellular processes (Table 1). Several metabolites derived from glucose metabolism activate ChREBP through covalent modification and allosteric regulation [27]. The covalent modification is mediated by the PPP intermediate Xu5P, which activates phosphatase A2, which in turn dephosphorylates ChREB, promoting its activation. Glc6P and Fru26BP may also directly bind to the ChREB protein, inducing a change from its inactive to active form [27]. ChREBP interacts with the basic helix-loop helix/leucine zipper protein Mlx to efficiently bind to ChoRE (carbohydrate-response element) sequences found in several genes that codify for target proteins [146]. It has been determined in breast carcinoma that the ChREBP protein levels are low in the first stages (I and II) of cancer development but increase once the malignant phenotype progresses (stages III and IV); thus, it has been considered to be a promising malignancy marker [147]. It is thought that ChREBP upregulates glycolysis since it increases the mRNA contents of several glycolytic proteins such as GLUTs (2,4,5), HPI, ALDO, GAPDH, and PYK-L (liver isoform) in normal (rat hepatocytes) [148] and PYK-LR (liver and red cell isoforms) cancer (HepG2) cells (Figure 1, Table 2), although the glycolytic flux has not been evaluated. In colon HCT116 cancer cells, ChREBP knockdown decreases glucose uptake and lactate production (Table 2), indicating a tight relationship between ChREBP and glycolysis in cancer cells. It is also known that ChREBP may be downregulated by AMP, ketone bodies, or cyclic AMP in hepatocytes: this metabolic regulation has not been examined in cancer cells [27].

### 2.2. Oncogenes

#### 2.2.1. c-MYC

The *MYC* proto-oncogene encodes the transcription factor c-MYC, the latter forming (with the Max protein) a heterodimer that binds the E-box sequences in target promoters [149]. Its canonical transcriptional roles in cellular processes are summarized in Table 1. At the metabolic level, c-MYC upregulates some glycolytic genes (*HKII*, *PFK-1*, *TPI*, *GAPDH*, *ENO*, *LDHA*, *MCT1*), leading to increased glycolytic fluxes (Figure 1, Table 2) in metastatic cancers such as mouse Eµ-Myc lymphoma cells and human Burkitt’s lymphoma P493 cells [64,65,66]. It has also been observed that in the latter cells, c-MYC stabilizes HIF-1α under normoxic conditions by blocking the HIF1α–von Hippel Lindau protein interaction for proteasomal degradation. Hypoxia alone or combined with hypoglycemia contributes to c-MYC proteasomal degradation in aggressive colon cancer HCT116 cells; in consequence, low c-MYC levels are found in xenograft tumor cells located away from blood vessels in hypoxic regions [150].

#### 2.2.2. RAS (HRAS and KRAS)

Both HRAS and KRAS (KRAS4A and KRAS4B) are members of the small GTPase RAS subfamily that is highly sensitive to GTP activation in cancer cells [151]. The MAPK/ERK signaling pathway involved in regulating cell division in response to growth factor stimulation is strongly regulated by RAS proteins (Table 1). While hypoxia increases the expression of nonmutated KRAS through protein kinase c-Src activation, hypoglycemia favors the expression of several KRAS mutants, with the G12D mutated protein being the most commonly expressed in cancer cells [68,152]. Indeed, mutations in HRAS (G12V, G13R, Q61R) and KRAS (G12D, G13D, Q61H) lead to the development of highly aggressive metastatic phenotypes [153].

Mutated KRAS^G13D^ overexpression increases the mRNA and protein contents of GLUT1 and HKII through the Raf/MRK/Erk/c-MYC and Pi3K/Akt pathways [68,69] in human colon HTC116 and DLD1 carcinomas (Figure 1, Table 2), as well as in HRAS-transformed embryonic NIH 3T3 fibroblasts and human breast epithelial MCF10A cells. As a consequence, the glycolytic flux in cancer and noncancer cells also increases (Table 2) [68,107,154]. In this regard, it has been suggested that mutated KRAS^G12V^ may induce HIF-1α stabilization, promoting HIF1α glycolytic target protein overexpression [69]. The suggested molecular mechanism involves KRAS^GV12^-induced ROS overproduction [155] acting as an OxPhos blocker [156]. ROS may oxidize cysteine residues of the PH catalytic sites, inhibiting their activity and preventing HIF-1α ubiquitination and degradation. KRAS^G13D^ also increases glycolysis through RAF/Mek/Erk/c-MYC pathway downstream activation [157].

### 2.3. Tumor-Suppressor Genes

#### 2.3.1. RB

The retinoblastoma protein (RB) belongs to the tumor-suppressor family involved in several cancer cell functions (Table 1) [26]. In its active state (as a monomer), RB binds to the E2F transcription factor family for cyclin-dependent kinase (CDK) transcriptional repression with concomitant cell cycle arrest. In cancer cells and biopsies, RB is usually inactivated by mutations or by nutritional stress through AMPK-dependent phosphorylation [158]; consequently, RB inactivation promotes cell cycle activation through E2F release (which in turn, induces the transcription of genes associated with the progression of G1 to an S phase in the cell cycle) and proapoptotic (BH3) protein depletion [159], favoring cancer onset and development. A few studies analyzing the role of RB in tumor glycolysis have been reported (Table 2). In human retinoblastomas, mutated RB overexpression increases ALDO and LDH activities (Table 2); however, the activities of other glycolytic enzymes (TPI, GAPDH, PGK, ENO, and PYK), including the controlling ones (HKII and HPI) [160] are severely decreased versus normal human adult retinas (Table 2, Figure 1). Unfortunately, glycolytic flux has not been evaluated to rigorously reveal the RB physiological role in glycolysis. It has been suggested that glycolytic activation by RB is mediated by c-MYC coactivation [161].

#### 2.3.2. PTEN

The phosphatase and tensin homolog (PTEN) shows high activity in its dimeric form and has three isoforms, the canonical PTEN, PTEN-Long, and PTEN-β [162]. The most frequent isoform found in tumors [163] is the canonical PTEN protein. PTEN is a member of the tyrosine phosphatase family [164] and is considered to be a tumor suppressor with growth and survival regulatory functions [165]. It is frequently found in its mutated (G129R) isoform in several metastatic and advanced cancers such as triple-negative breast cancer, glioblastoma, and prostate carcinomas [164]. Its canonical functions are listed in Table 1. In metastatic prostate cancer cells, PTEN repression using siRNAs increases HKII protein content as well as glucose consumption and lactate production (Figure 1, Table 2). The molecular mechanisms involved have not been elucidated, but it has been proposed that PTEN blocks HKII mRNA translation through the inactivation of signaling pathways related to protein synthesis such as AKT/mTORC1/4EP1 [72]. Then, it seems that PTEN deficiency may reprogram glucose metabolism in cancer cells. Increased PTEN levels decrease glucose uptake and increase mitochondrial biogenesis and drug resistance in several cancer cells (Table 2) [166]. The molecular mechanisms associated with the PTEN regulation of tumor glycolysis require further investigation.

### 2.4. Protein Kinases

#### 2.4.1. JNK

JNK (JUN N-terminal kinase, renamed MAPK8 (mitogen-activated protein kinase 8)) is a serine/threonine protein kinase that shows activity in its dimeric form. Ten JNK isoforms have been described [167]. JNK’s most known isoforms are JNK1 (JNK1α1, JNK1β1, JNK1α2, JNK1β2), JNK2 (JNK2α1, JNK2β1, JNK2α2, JNK2β2), and JNK3 (JNK3α1, JNK3α2). JNK phosphorylates the JUN protein, which together with the c-FOS protein forms the AP-1 transcription factor. MAPK kinase-4 and MAPK kinase-7 are specific activators of JNK canonical pathways (Table 1) [36]. In liver carcinoma, the JNK2 isoform upregulates the PARP14 protein for glycolysis activation [168]. PARP14 overexpression blocks JNK1. JNK1 phosphorylates and activates PYKM2 and glycolysis. Then, PARP14 downregulation leads to JNK1 activation, PYKM2 phosphorylation, and glycolysis depression (Table 2) [74].

#### 2.4.2. mTOR

The mammalian target of rapamycin (mTORα and mTORβ) belongs to the phosphatidylinositol 3-kinase-related kinase family [37], with the most common being the mTORα isoform [169]. When it binds to several proteins (i.e., RAPTOR, mLST8, RICTOR, mSIN1, mLST8), it forms the active core components of two different protein complexes called mTOR complex 1 (mTORC1) and complex 2 (mTORC2). Hypoxia (0.1% O_2_) inhibits mTOR function by increasing the level of the mTOR canonical inhibitor TSC1 or by upregulating the *REDD1* gene involved in TSC1/2 complex activation [170]. In normoxia, mTORC1 is found in its active isoform, increasing cell proliferation in several human carcinomas (breast, colorectal, glioblastoma, lymphoma, and prostate) [171]. In parallel, mTORC1 activation increases GLUT1 and PFK1 mRNA levels as well as glucose consumption in cervix HeLa and leukemia MOLM-14 cells (Figure 1, Table 2). Apparently, the mTOR-induced glycolysis activation mechanism is linked to HIF-1α activation [75]. In addition, mTORC1 upregulates PPP through the activation of the sterol regulatory element-binding protein (SREBP), a transcriptional factor that increases the mRNA levels of Glc6PDH and ribulose-5-phosphate epimerase in HeLa cells (Table 2). Unfortunately, PPP flux has not been determined, and it has not been ascertained whether mTORC1 is physiologically involved in the regulation of this pathway.

## 3. Transcriptional Regulators of Tumor Oxidative Phosphorylation (OxPhos) in Cancer Cells

### 3.1. Transcription Factors

#### 3.1.1. HIF-1α

**Figure 2:**
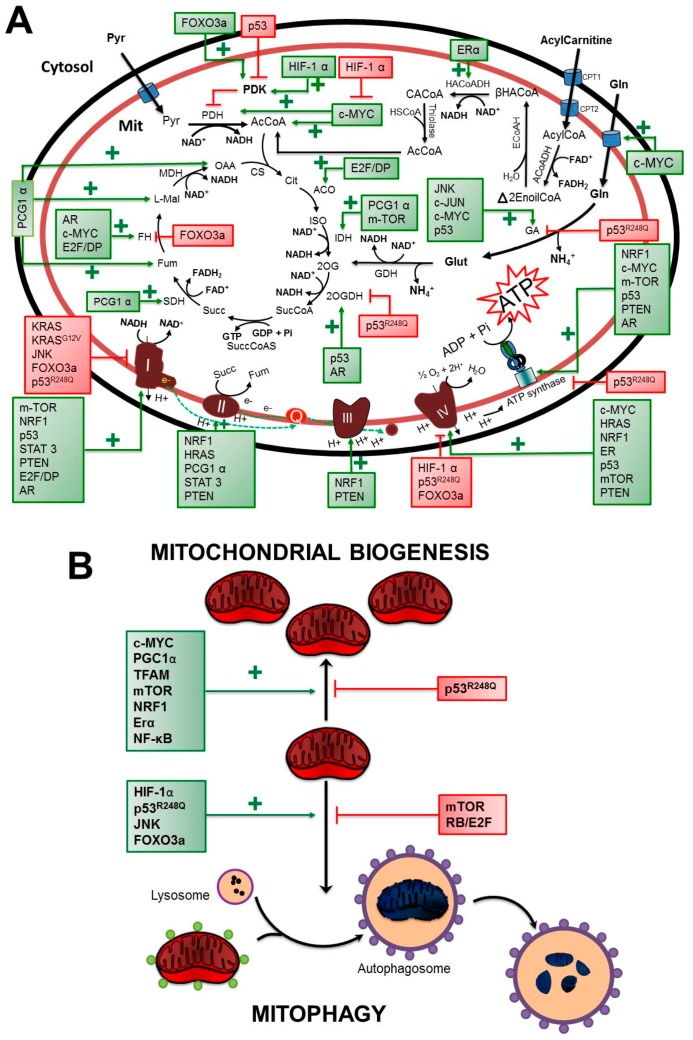
Transcription regulators that modulate (**A**) OxPhos and (**B**) mitochondrial biogenesis/mitophagy in cancer cells. Color codes are as in Figure 1. Abbreviations: 2-OG, 2-oxoglutarate; 2OGDH, 2 oxoglutarate dehydrogenase; ACO, aconitase; Cit, citrate; CS, citrate synthase; cyt c, cytochrome c; FH, fumarate hydratase; Fum, fumarate; GA, glutaminase; GDH, glutamate dehydrogenase; Gln, glutamine; Glut, glutamate; HACoADH, β-hydroxyacyl-CoA dehydrogenase; IDH, isocitrate dehydrogenase; Iso, isocitrate; Mal, malate; MDH, malate dehydrogenase; Mit, mitochondria; OAA, oxaloacetate; PDH, pyruvate dehydrogenase complex; Pyr, pyruvate; Q, quinone; SCoAS, succinyl CoA synthase; SDH, succinate dehydrogenase; Succ, succinate.

As previously indicated, HIF-1α is a key TF that upregulates glycolysis and downregulates mitochondrial function under hypoxic stress (0.1–1% O_2_ for 24–48 h) [81]. HIF1-α downregulates OxPhos in human Burkitt’s lymphoma by increasing the PDK mRNA content [80]. This mitochondrial matrix kinase phosphorylates and inhibits PDH complex activity, and hence the flux through the Krebs cycle decreases (Figure 2A, Table 3), which most likely affects OxPhos. In hypoxic Hep3B, cervix HeLa, colon HCT116, and lung A594 cancer cells, HIF-1α also induces decreased COX4-1 protein contents (Figure 2A, Table 3). The most common interpretation of these types of observations is that the cellular function associated with the measured protein (or mRNA) is also modified. In this regard, it is worth noting that the variation (increment/decrement) in the mRNA levels of many TF target enzymes/transporters (Figure 1 and Figure 2) frequently shows no strict correlation with their respective protein contents, activities, and (importantly) corresponding metabolic pathway fluxes or biological functions. To avoid the overinterpretation of variations in mRNA levels, it is advisable to assess their respective functions [172].

Thus, it has been stated that HIF-1α downregulates respiratory chain activity and OxPhos because it modifies the mRNA level of one of its many components (Figure 2A, Table 3) [81]. However, it is unknown how much COX subunit 4-1 has to be decreased in order to affect the redox and H^+^ pumping activity of respiratory complex IV. Moreover, COX activity and OxPhos flux have not been directly determined [81] to assess whether HIF-1α indeed may downregulate the mitochondrial function in cancer cells.

HIF-1α also downregulates OxPhos in metastatic cells, activating several autophagy genes, such as *BNIP3*, which in turn increases mitochondrial digestion [82] (Figure 2B, Table 3).

#### 3.1.2. p53, Wild-Type, and Mutant Isoforms

In normoxia, the p53 wild-type isoform favors mitochondrial metabolism by increasing (a) the mRNA content encoding SCO2c, a chaperone protein required for correct COX and GA assembly in HCT116 and HepG2 tumor cells, respectively [85,86]; (b) the protein levels of ND1, COX4, 2OGDH, GA, and ATPS in p53 overexpressing HeLa cells [52]; (c) the AIF (apoptosis-inducing factor) and Parkin (a RBR E3-ubiquitin protein ligase) levels involved in ND1 and PDH activation in nonsmall-cell lung H1299 cancer and large-cell lung H460 cancer cells [87,88]; and (d) the mitochondrial membrane potential (Δψ_m_) and the fluxes of total oxygen consumption, OxPhos, and glutaminolysis in colon HCT116, H460, and p53 overexpressing HeLa cells (Table 3). In addition, wild-type p53 represses the transcription of PDK2, which is involved in PDH inactivation in MCF-7 and HCT116 cells [84]. Under severe and prolonged hypoxia (0.1% O_2_, 24 h), the protein contents of several OxPhos enzymes, such as COX4, 2OGDH, and ATPS, as well as OxPhos flux, are markedly diminished in p53 overexpressing HeLa cells in comparison to p53-lacking cells (Figure 2A, Table 3).

On the contrary, in mutant p53^R248Q^-expressing HeLa cells, mitochondrial metabolism is depressed under both normoxia and hypoxia conditions, which correlates with an active mitochondrial digestion and attenuated mitochondrial biogenesis [52] (Figure 2A, Table 3).

A regulatory role of TIGAR (TP53-induced glycolysis regulatory phosphatase) in OxPhos has not been established. However, in TIGAR-overexpressing T98G and LNT-299 glioma cells, the total oxygen consumption and OxPhos flux increase by 10–50%, suggesting the upregulation of mitochondrial function (Table 3).

#### 3.1.3. PGC1α

This TF coactivates and increases the transcriptional activity of NRF-1 (nuclear respiratory factor 1; see next subsection), leading to mitochondria biogenesis onset and respiration stimulation in immortalized mouse C2C12 fibroblasts [173]. PGC1α overexpression also increases the (i) mRNA contents of SDH, IDH3, and cytosolic aspartate aminotransferase; (ii) β-oxidation flux; (iii) the contents of the Krebs cycle intermediates OAA, fumarate, and L-malate; and (iv) ATP intracellular content in metastatic prostate cancer PC3 cells versus nonexpressing PGC1α cells (Figure 2, Table 3).

#### 3.1.4. NRF-1

NRF (nuclear respiratory factor-1) is a family of TFs constituted by NRF1α, NRF1β, and NRF1γ. Under normoxia, metastatic and low-metastatic cancers (bladder-urothelial, breast, and colon adenocarcinomas; head and neck squamous cell carcinoma) maintain high mRNA levels of NRF-1 [174]. A high NRF-1 protein level is linked to an increment in the transcription of nuclear genes encoding some subunits of several respiratory chain complexes [90,91] and *ATPS* in cervix HeLa cancer cells (Figure 2; Table 3). It has been suggested that NRF-1 may also induce the expression of several components of mtDNA transcription and replication machinery as well as the mitochondrial heme biosynthetic pathway [175]. NRF-1 overexpression induces a malignant phenotype in epithelial mammary gland MCF10A cells and increases the total oxygen consumption as well as intracellular ATP levels, multicellular spheroid formation, cell migration, and cellular invasion abilities. NRF-1 knockdown in breast cancer MDA-MB-231 cells brings about a significant decrease in all mitochondrial function parameters, including total oxygen consumption and ATP levels [19]. Under hypoxia, NRF-1 is degraded through the ubiquitination system, resulting in an enhanced glycolytic rate, metabolic reprogramming, and a protumor immune response. NRF-1 degradation induced by hypoxia promotes some cancer cells becoming more susceptible to apoptosis [176]: this interesting observation deserves further study.

#### 3.1.5. NF-κB

This TF is constitutively active in many different types of cancer cells, leading to the expression of genes involved in cancer progression (Table 1). Overexpression of the ReIA subunit of NF-κB activates nonmutant p53 protein expression (see p53 in Section 3.1.2 above). Thus, NF-κB may also be described as a promitochondrial TF in a process mediated by nonmutant p53. In mouse skeletal muscle, the NF-κB regulator kinase IKKα and the RelB subunit stimulate mitochondrial biogenesis by activating PGC1-α transcription [177].

#### 3.1.6. TFAM

Cancer canonical functions of the transcription factor A are listed in Table 1. TFAM is required for mtDNA stability: its downregulation decreases mtDNA copy numbers and enhances sensitivity to cisplatin and doxorubicin in liver Hep-2, nasopharyngeal HNE2, and lung A549 carcinoma cells, promoting ROS-induced apoptosis.

#### 3.1.7. STAT3

Once STAT3 is activated by phosphorylation, this TF interacts with the mitochondrial import protein GRIM-19 [178] and is translocated from the cytosol to the mitochondria in both noncancer (heart and liver cells, lymphocytes) and cancer (HRAS-transformed human bladder T24 carcinoma) cells. Once inside the mitochondria, STAT3 directly interacts and increases the activity of ND1 and SDH, as well as increases OxPhos flux (Figure 2A, Table 3). Mitochondrial STAT3 activation has not been characterized in mitochondria from cancer cells. However, in isolated heart mitochondria from mice subjected to ischemia (i.e., hypoxic condition), STAT3 protects ND1 and SDH activity from ischemic damage, acting as an ROS scavenger-like molecule by decreasing ROS production from both respiratory complexes I and II [179]: the precise mechanism of this relevant STAT3 action remains to be elucidated, but it might involve increased electron transfer efficiency between respiratory complexes and/or the activation of antioxidant enzymes.

#### 3.1.8. PTEN

In several human glioma cells, PTEN overexpression increases the protein content levels of all respiratory chain complexes and OxPhos flux (Figure 2, Table 3), as well as the protein content of several promitochondrial TFs (such as PGC1α and p53) that upregulate the genes of mitochondrial proteins. However, in normal cells (mouse fibroblasts), PTEN promotes the opposite effect: it significantly diminishes the protein content and activity of respiratory chain complexes and concomitantly the OxPhos flux by 30–60% [115]. The mechanisms involved in PTEN–mitochondrial upregulation in cancer cells have not been elucidated.

#### 3.1.9. FOXO3a

The activation of FOXO3a in DLD-1 colon cancer (Table 3) and immortalized epithelial RPE-hTERT cells is associated with mitochondrial function inactivation. FOXO3a activation decreases total oxygen consumption in cancer cells because PDK4 is upregulated, and several genes of the Krebs cycle (*FH*) and OxPhos (*ND1, COX*) are downregulated (Figure 2, Table 3). In Rat-1 fibroblasts, FOXO3a induces mitophagy through BNIP3 overexpression [180].

#### 3.1.10. E2F

E2F also upregulates several OxPhos genes in cancer cells. The E2F/DP complex binds to the promoter region of *ND1* and Krebs cycle (*ACO, FH*) genes in sarcoma osteogenic SAOS-2 cells, increasing their mRNA levels [97]. On the other hand, the depletion of DP protein leads to E2F inactivation, resulting in abnormal mitochondrial shapes and decreased Δψm (Table 3).

#### 3.1.11. AR

The activation of the androgen receptor (AR) with a synthetic androgenic agonist (R1881) in the human androgen-dependent prostate cancer cells LNCaP and LAPC4 promotes an increase in mRNA levels of Krebs cycle enzymes (FH, 2OGDH), ATPS, and ND1, as well as OxPhos flux (Figure 2A, Table 3). This enhancement in the mitochondrial metabolism has been associated with prostate cancer proliferation and progression [59].

#### 3.1.12. ERs

ERα (ERα, ERα46, and ERα36) and ERβ (ERβ, ERβ2, ERβ4, and ERβ5) are homodimers/heterodimer-forming estrogen receptors. ERs belong to the steroid/nuclear receptor superfamily of TFs that is involved in the expression of genes associated with the proliferation and regulation of female sexual phenotypes [29,181]. Regarding mitochondrial metabolism, the interaction between ERα and its natural ligand 17β-estradiol promotes the transcription of NRF-1, which in turn activates mitochondrial biogenesis and mitochondrial oxygen consumption in low-metastatic breast MCF-7 and lung H1793 cancer cells (Table 3). Therefore, ERα has been considered to be a positive but indirect OxPhos TR (Figure 2, Table 3). ERβ bound to 17β-estradiol is translocated into mitochondria to interact with mtDNA, increasing mitochondrial COX mRNA levels [98]. Transcriptional regulation by ERβ of other mtDNA-encoded OxPhos genes has not been analyzed. In mitochondria of breast MCF-7 and ERα-transfected MDA-MB-231cancer cells, a direct interaction between ERα and β-hydroxyacyl-CoA dehydrogenase (HACoADH), an enzyme involved in free fatty acid (FFA) β-oxidation, has been described. This interaction increases enzyme activity and perhaps FFA oxidation in cancer cells, but β-oxidation flux has not been evaluated [99].

#### 3.1.13. PPARs

The peroxisome proliferator-activated receptors PPAR-α, PPAR-δ/β, and PPAR-γ belong to the TF-activated nuclear receptor superfamily. PPAR-α is expressed in brown adipose tissue, the liver, kidneys, and the heart, and this isoform is the main fatty acid β-oxidation inducer. PPAR-δ/β is ubiquitously expressed in most tissues (i.e., the brain, kidneys, heart, and liver) and also activates fatty acid β-oxidation [182]. In turn, PPAR-γ activates adipocyte differentiation and regulates cellular lipogenesis [183]. In cancer (HeLa cervix, 143B osteosarcoma, MDA-MB-231 breast) cells [101] and hepatocytes [184], the activation of PPAR isoforms with either bezafibrate or tetradecylthio acetic acid increases the activity of several mitochondrial proteins, such as carnitine palmitoyl transferases (CPT-I and CPTII), citrate synthase (CS), and cytochrome oxidase (COX), as well as increases by 40% cellular ATP levels and mitochondrial membrane potential. Although the molecular mechanism has not been determined, it has been proposed that PPAR activation is linked to the PGC1α and mTOR signaling pathways [101,184].

### 3.2. Oncogenes

#### 3.2.1. c-MYC

**Figure 3:**
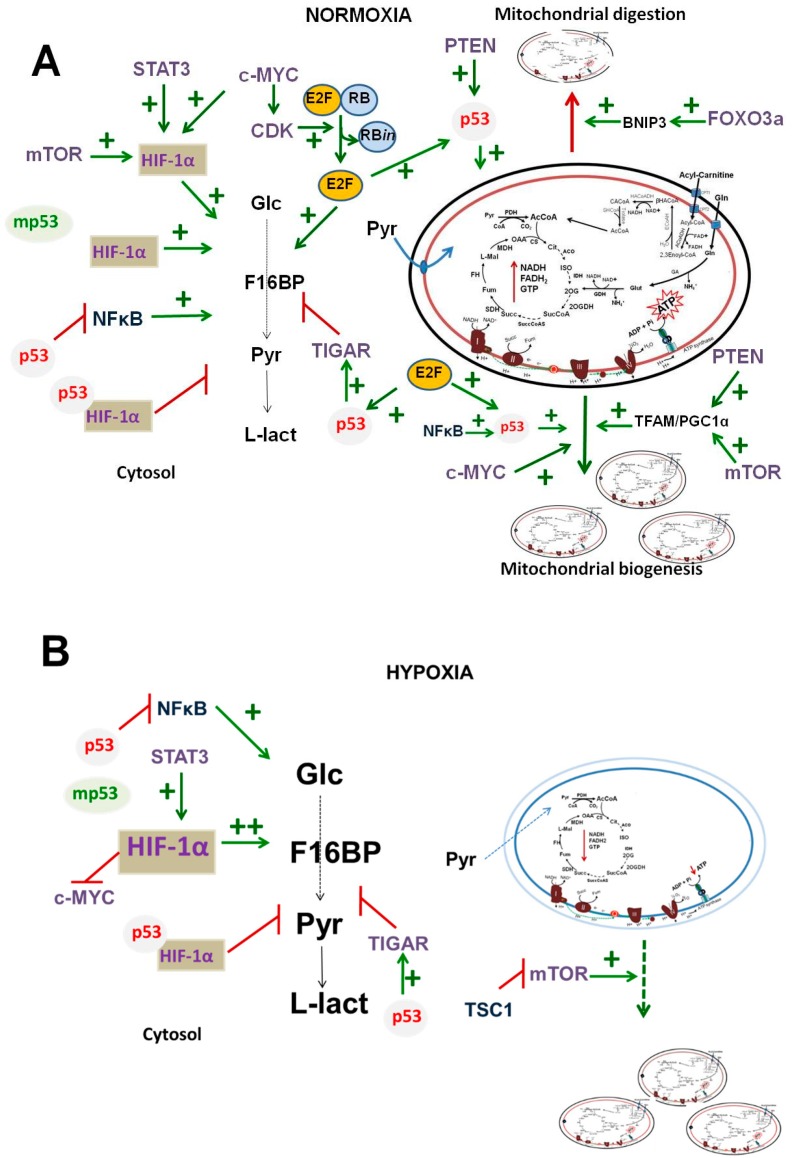
Transcription regulator interplay, acting on energy metabolism under (**A**) normoxia and (**B**) hypoxia. Abbreviations as in Figure 1 and Figure 2; mp53, mutant p53; RB*in*, inactive RB protein.

Further, c-MYC upregulates OxPhos in several cancer cell lines because it (i) increases the mRNA content of several genes involved in mitochondrial biogenesis (*MIEFs, TFAM, mitoRBs*), mitochondrial stability (*BCS1L, COX15*), and OxPhos (*COX4, PDH, FH, ATPS, GA,* glutamine transporters *SLC38A5* and *SLC1A5*); (ii) increases the activity of GA; and (iii) enhances mitochondrial acetyl-CoA levels, indicating active pyruvate, ketone body, glutaminolysis, and FFA oxidation versus nonactivated c-MYC cells (Figure 2A, Table 3). Under normoxia, a high level of endogenous c-MYC is found in several cancer cell types: c-MYC also upregulates glycolysis. Glycolysis enhancement maintains elevated levels of the biosynthetic precursors required for cell growth, whereas increased mitochondrial activity is required for ATP supply. Hypoxia (0.1–1% O_2_) stabilizes HIF-1α, inhibiting c-MYC transcriptional activity [185]; in consequence, cellular functions such as cell cycle and OxPhos, which are highly dependent on c-MYC under normoxia, are severely affected under hypoxia (Figure 3B).

#### 3.2.2. RAS (HRAS and KRAS)

Nonmutated HRAS overexpression increases the protein contents of SDH and COX4, which correlates with a substantial increment in the OxPhos flux in transformed versus nontransformed mouse 3T3 fibroblasts (Figure 2A, Table 3). In contrast, the G12V and Q61L HRAS mutants expressed in the same mouse 3T3 fibroblasts stimulate glycolysis flux and decrease mitochondrial activity, indicating that HRAS mutations promote a gain in glycolytic function and a loss in OxPhos function (Table 3). However, human bronchial epithelial cells transformed by HRAS^G12V^ mutations favor glucose oxidation to pyruvate, which in turn is actively oxidized to mitochondrial 2-OG and hence to glutamate, glutamine, and aspartate, correlating with high total oxygen consumption and respiratory activity [110]. It has been suggested that these metabolic differences could be attributed to differences in genetic backgrounds between transformed and untransformed cells [186]. However, to find mechanistic explanations, differences in the interactions of native versus mutant HRAS with other TRs should also be considered, as they have already been documented in native and mutant p53 [50,52].

Regarding KRAS, both the nonmutated (transformed mouse 3T3 fibroblasts) and mutated (KRAS^G12V^-transformed human embryonic kidney 293 cells) forms promote a substantial decrement in the protein content and activity of ND1, an essential subunit of respiratory complex I, versus their respective nontransformed cells. Lowered ND1 content and activity correlates with diminished oxygen consumption in both transformed cell types. Although nonmutant KRAS cells maintain higher oxygen consumption rates (two times) versus mutant KRAS cells (Table 3), a strict mechanistic conclusion is still not possible because of the different genetic backgrounds of both transformed cell types [186].

### 3.3. Tumor-Suppressor Genes

#### RB

The inactivation of RB favors mitochondrial metabolism as well as cell proliferation in MEFs [176]. In RB-knockout MEFs, the mRNA contents of the total glutamine transporter (*ASCT2*) and *GA-1* increase, with a concomitant higher glutamine uptake and OxPhos flux versus wild-type MEFs, perhaps through a mechanism associated with E2F activation [187]. In breast MDA-MB-231 cancer cells, the inactivation of RB by tigecycline increases intracellular ATP content (Table 3). In human osteosarcoma Saos2 and U2OS cells, RB may indirectly activate OxPhos, preventing mitophagy onset by blocking the autophagy-positive regulator BNIP3 [188].

### 3.4. Protein Kinases

#### 3.4.1. JNK

In cancer cells, JNK translocation from the cytosol into the mitochondria decreases ND1 activity through JNK-mediated ND1 phosphorylation and inactivation: total oxygen consumption and OxPhos flux are also attenuated (Figure 2, Table 3). These observations suggest that phosphorylation/dephosphorylation may be a relatively novel regulation mechanism of the respiratory chain complexes and mitochondrial matrix enzymes, which deserves further research. JNK also downregulates OxPhos in neuroblastoma SH-SY5Y cells by inducing the overexpression of the positive mitophagy modulator BNIP-3, hence triggering mitochondrial digestion [189]. Indeed, the mitophagy inducer BNIP3 is a target of several TFs.

JUN belongs to the early response TF (AP-1) family, and it is activated by JNKs [190]. After activation, JUN binds to the proto-oncogene FOS, upregulating the transcription of genes involved in cell cycle progression and apoptosis (Table 1). In several metastatic breast cancer cells, JUN directly binds to the GA promoter region and upregulates its expression (Figure 2, Table 3). Although GA is an enzyme usually found in the mitochondrial matrix, it has been established that GA also plays a role as a nuclear transcription factor inhibiting the PPARγ signaling pathway [114]. Unfortunately, there are no studies analyzing the regulatory role of JUN in OxPhos in cancer cells.

#### 3.4.2. mTOR

In breast MCF-7 cancer cells, mTOR activates mitochondrial biogenesis by increasing TFAM (Figure 3A), mitochondrial ribosomal proteins, and some components of respiratory complex I (NDUFAF2, NDUFAF4, and NDUFS6) and ATPS (ATP5D, ATP5L, ATP5G1, ATP5O) [119]. In addition, mTORC1 increases (by two times) the transcription of several nuclear-encoded genes of mitochondrial proteins (ATPS, COX, IDH3) through a mechanism mediated by Yin Yang 1 and PGC-1α in cancer cells [120]. Further, mTOR also suppresses mitophagy through ATG1 dephosphorylation and inactivation in cancer cells (Figure 2, Table 3).

### 3.5. Plasma Membrane Receptors

#### Notch 1

The *NOTCH* gene codes for at least four highly conserved type I plasma membrane receptors (TIMRs) that selectively bind Notch 1 to Notch 4, negative (Numb) and positive (DXT1) modifiers of some signaling pathways, and TFs such as CLS (an acronym for CBF-1/RBPJ-κ in *Homo sapiens*/*Mus musculus*, suppressor of hairless in *Drosophila melanogaster*, and Lag-1 in *Caenorhabditis elegans*) [191]. During transactivation (i.e., once Notch recognizes and binds its ligand), two successive proteolytic cleavages occur, in which the intracellular domain of Notch (NICD) is translocated from the cell membrane to the nucleus. There, NICD specifically binds to the DNA-binding protein CSL for gene expression regulation [38]. Notch 1 inactivation has been related to a decrement in the oxygen consumption flux in breast MDA-MB-231 carcinoma (Table 3): that is, Notch 1 may positively modulate mitochondrial function. The proposed mechanism is related to NF-κB activation mediated by AKT and IKK phosphorylation induced by Notch1 [122]: NF-κB in turn promotes mitochondrial biogenesis (see NF-κB section). Unfortunately, OxPhos flux and protein contents have not been directly evaluated in noncancer or cancer cells when Notch1 expression is varied.

## 4. Overview of TR Interplay and Action in Cancer Glycolysis and OxPhos

### 4.1. p53

Under either normoxia or hypoxia, nonmutant p53 directly (a) binds to HIF1-α, blocking its transcriptional activity, and (b) induces the overexpression of TIGAR to deter glycolysis. Under normoxia, p53 favors OxPhos, which provides energy to sustain tumor development (Table 2A), but under hypoxia, OxPhos is severely depressed by nonmutant p53, and glycolysis becomes the predominant ATP supplier [50]. E2F favors OxPhos because this TF directly activates nonmutant p53 [192] (Figure 3A). Independently of oxygen availability, mutant p53 (p53^R248Q^) cannot bind to HIF1-α, which then exerts its canonical function without restrictions, thus increasing glycolytic flux. Mutant p53 stimulates mitochondrial digestion (Figure 2B), and the Warburg effect is favored in cancer cells [52] (Figure 3).

NF-κB increases glycolytic flux under both normoxia and hypoxia. However, in the presence of nonmutant or mutant p53, NF-κB activation is suppressed, which leads to glycolysis depression. In normoxia, NF-κB overexpression stabilizes p53, favoring mitochondrial biogenesis and indirectly OxPhos (Figure 3A).

### 4.2. c-MYC

Normoxia stabilizes the c-MYC protein, which promotes both glycolysis and OxPhos activation because c-MYC (a) induces HIF-1α protein stabilization; (b) increases the expression of *CDK*, which triggers RB phosphorylation, which in turn promotes E2F release and glycolysis activation; and (c) increases mitochondrial biogenesis. Thus, c-MYC may be considered to be a proglycolytic and promitochondrial TF under normoxic conditions (Figure 3A). On the contrary, hypoxia promotes c-MYC degradation; therefore, the high glycolysis observed under an oxygen limitation is mostly due to HIF-1α activation (Figure 3B).

### 4.3. mTOR

Under normoxia, mitochondrial metabolism in cancer cells may be favored because mTOR triggers mitochondrial biogenesis through TFAM and PGC1-α activation [120]; in addition, mTOR may upregulate glycolysis through HIF-1α activation [75] (Figure 3A). Under hypoxia, mTOR is inactivated by the increment of its canonical inhibitor TSC1, contributing to general hypoxia-induced OxPhos impairment. Thus, the enhanced glycolysis induced by hypoxia is mainly sustained by HIF-1α activation (Figure 3B).

### 4.4. FOXO3a

Under normoxia, FOXO3a overexpression favors BNIP-3-induced mitophagy in cancer cells [189,193]. However, the total mitochondria content depends on both mitochondrial biogenesis as well as mitophagy. In normoxia, mitochondrial biogenesis is activated by c-MYC, PGC-1α/NRF-1, and/or RB; therefore, due to the stabilization of all of these TFs and oncogenes, mitochondrial biosynthesis prevails over mitochondrial digestion (Figure 2B and Figure 3A).

### 4.5. RAS

Nonmutant HRAS and nonmutant p53 favors OxPhos flux, whereas mutant p53 and mutant HRAS induce or favor the Warburg phenotype, i.e., there is induction of glycolysis and attenuation of OxPhos (Figure 3). In turn, both mutant and nonmutant KRAS favor the Warburg phenotype in cancer cells. Thus, it can be predicted that tumors with high levels of mutated HRAS, KRAS, or p53 will very likely develop and show a Warburg phenotype.

### 4.6. STAT3

Under normoxia or hypoxia, this TF stabilizes HIF1-α, thus increasing glycolytic flux (Figure 3).

## 5. Concluding Remarks

Energy metabolism pathways play an important dual role in cancer cells. They regulate the supplies of ATP and are precursors for energy-demanding (proliferation, differentiation, migration, invasion, colonization, ion homeostasis) and anabolic (biosynthesis of nucleic acids, proteins, phospholipids, cholesterol) processes. The energy metabolism pathways are in turn regulated by oxygen and substrate (glucose, glutamine, fatty acids) availability through the action of multiple TRs.

In cells localized close to blood vessels (i.e., under normoxia and normoglycemia) within solid tumors, glycolysis mainly provides precursors for the biosynthesis of proteins (3PG, Pyr), nucleic acids (R5P), glycogen (Glc6P), and phospholipids (glycerol-3-phosphate) required for cell proliferation. In turn, OxPhos provides most of the ATP required for cell proliferation and other cell functions, as well as anabolic precursors (citrate, 2-OG).

In cells localized far away from blood vessels (under hypoxia and hypoglycemia) within solid tumors, energy metabolism reprogramming is triggered, in which glycolysis becomes the main ATP supplier and OxPhos is depressed [194]. It is worth noting that the majority of studies on cancer TRs and energy metabolism regulation have been carried out exclusively under normoxic conditions, except for those whose stabilization/overexpression required hypoxia, such as HIF-1α or p53. An analysis of the interplay between TRs and energy metabolism under hypoxia will be relevant in future studies because their regulation mechanisms in energy metabolism pathways in cancer cells very likely change, leading to metabolic reprogramming, increased drug resistance, cancer stem cell enrichment, and metastasis.

## Figures and Tables

**Table 1:** Transcription regulators involved in several cancer cell functions.

Transcription Regulator (Protein)	Intracellular Localization	Canonical Cellular Process Target	Refs.
**Transcription Factors**			
HIF-1α	Nuclei	Angiogenesis, erythropoiesis, cellular proliferation, survival, vascular remodeling, tumorigenesis, invasion, metastasis	[16]
p53	Nuclei	Cell cycle inhibition, apoptosis onset, antioxidant response, DNA damage repair systems, senescence, mitophagy	[17]
PGC-1α	Nuclei	Mitochondrial biogenesis and oxidative metabolism	[18]
NRF-1	Nuclei	Expression of nuclear genes required for mitochondrial metabolism	[19]
NF-κB	Nuclei	Immune response, proliferation, apoptosis and angiogenesis suppression, metastasis	[20]
TFAM	Mitochondria	Cell cycle regulator, metastasis progression	[21]
STAT3	Nuclei	Inhibition of immune activation against tumor cells, cancer progression	[22,23,24]
FOXO	Nuclei	Regulator of cell proliferation, apoptosis, invasion, metastasis	[25]
E2F	Nuclei	Cell proliferation, angiogenesis	[26]
ChREBP	Nuclei	Regulator of glucose metabolism and lipogenesis	[27]
AR	Nuclei	Regulator of development and function of male reproductive system and male phenotype	[28]
ER	Nuclei	Regulator of development and function of female reproductive system and female phenotype	[29]
PPARs	Nuclei	Regulator of lipid metabolism	[30]
p53-Induced Phosphatase			
TIGAR	Cytosol	Cancer chemoresistance	[31,32]
**Oncogenes**			
c-MYC	Nuclei Cytosol	Cell cycle regulation, apoptosis, cellular transformation	[33]
HRAS and KRAS		Metastasis and aggressive phenotype	[34]
Tumor Suppressor			
PTEN	Nuclei and cytosol	PI3K/AKT pathway blocking	[35]
**Protein Kinases**			
JNK	Cytosol	Cell proliferation, differentiation, development, inflammatory response, apoptosis, malignancy, tumorigenesis	[36]
mTOR	Cytosol	Energy metabolism reprogramming, nutrient sensor	[37]
**Plasma Membrane Receptors**			
Notch1	Nuclei	Regulator of gene expression	[38]

**Table 2:** Transcription regulators of cancer glycolysis.

Transcription Regulator (Protein)	Cancer Cell	Target	Measured Parameter	Variation	Refs.
**Transcription Factor**					
HIF-1α	Human U87 glioma	GLUT3, ALDO-A	mRNA content	Up ~2 times	[47]
HIF-1α (Hypoxia)	Human cervix HeLa; human liver HepG3B; human lung A549; human breast MCF-7 and MDA-MB-231; human colon LS174 and BE; human renal clear cell RCCA carcinomas; human glioma U87; mouse HepaC1 and HepaC4 hepatomas	GLUT1, HKI, HKII, PFK-L, ALDO-A, TPI, GAPDH, PGK1, ENO, PYK-M2, LDH-A, MCT4, GS, PGM	mRNA content	Up ~1.1–30 times	[5,39,40,41,42,43,48,49]
			Protein content	Up ~2–10 times	
			Glycolysis flux	Up ~3–6 times	
			Glycogen content	Up ~1.7–26 times	
HIF-2α (Hypoxia)	human breast cancer MDA-MB-231, MDA-MB-468	PGK1, PGM-1, PYKM, LDH-	mRNA content	Unchanged	[44]
	Human renal 786-0 carcinoma	GLUT1	mRNA content	Up ~ 2 times	[45]
			Protein content	Up ~ 2 times	
p53 (Normoxia)	Human Saos-2 sarcoma, human cervix HeLa carcinoma	GLUT1, GLUT3, GLUT4	Protein content	Down 40–70%	[50,51]
			Glycolytic flux	Unchanged	
	Mutant p53 ^R248Q^ cervix HeLa carcinoma	GLUT1, GLUT3, HKI, HKII	Protein content	Up ~2–3 times	[50]
			Glycolysis flux	Up ~2 times	
p53 (Hypoxia)	Human cervix HeLa carcinoma	GLUT1, GLUT3	Protein content	Up ~1–2.5 times	[50]
			Glycolytic flux	Down ~30%	
	Mutant p53 ^R175H^, ^R248Q^ and R273H human cervix HeLa carcinoma; human lung H1299 carcinoma	GLUT1, GLUT3, HKI, HKII	Protein content	Up ~1–2 times	[52,53]
			Glycolysis flux, ECAR	Up ~1.5–2 times	
TFAM (Decrement by 30–70%)	Human lung A549 and H460 carcinomas		Glycolytic flux	Down 30–70%	[54]
STAT3	Human liver HepG2 and Hep3B carcinomas; human HCV virus-related hepatocarcinoma	GLUT1, HKII	mRNA content	Up ~1.4 times	[55,56,57]
			Protein content	Up ~1.3 times	
			Glucose consumption	Up ~1 time	
			Lactate production	Up ~1.6 times	
E2F	Rat rhabdomyosarcoma	Fetal-type PFK-2/F2,6BPase	mRNA content	Not reported	[58]
AR	Human prostate LNCaP and LAPC4 carcinomas	HKII, PFK-P, ENO, PGK	mRNA content	Up ~1–3 times	[59,60]
			ECAR	Up ~2.5–5 times	
ChREBP	Human hepatocarcinoma HepG2	PYK-LR	mRNA content	Up ~2 times	[61]
ChREBP (SUPRESSION)	Human colon HCT116 carcinoma		Glucose uptake	Down ~50–60% Down ~60–70%	[62]
			Lactate production		
**p53-Induced Phosphatase**					
TIGAR (Normoxia)	Human bone U20S osteosarcoma	Fru-2,6-BP2	Metabolite content	Down ~70–80%	[31]
TIGAR (Hypoxia)	Human ovarian A2780 and SW48 carcinomas	HKII	Activity	Up ~1.4 times	[63]
**Oncogene**					
c-MYC	Human Burkitt’s P493 lymphoma; mouse Eµ-Myc lymphoma	HKII, PFK-1, GAPDH, TPI, ENO, LDH-A, MCT1	mRNA content	Up ~1–17 times	[64,65,66,67]
			Protein content	Up ~2–3 times	
			Glycolysis flux	Up ~1.5–3 times	
KRAS	Mutant KRAS^G13D^ human colon HTC116, DLD1 carcinomas	GLUT1, HKII	mRNA content	Up ~1.7–5 times	[68,69]
			Protein content	Up ~3–5 times	
			Glycolysis flux	Up ~2 times	
**Tumor Suppressors**					
Inactive RB	Human retinoblastoma biopsies	ALDO, LDH	Activity	Up ~1.2–10 times	[70]
		HKII, HPI, TPI, GAPDH, ENO, PGK, PYK	Activity	Down ~20–80%	[71]
PTEN (Suppression)	Human prostate DU-145, 22Rv1 carcinomas	HKII	Protein content	Up ~1.5 times	[72]
			Glucose consumption	Up ~1.2–1.4 times	
			Lactate production	Up ~1.2 times	
PTEN (Overexpression)	Human ovarian carcinoma cells A2780 and SKOV-3		Glucose consumption	Down 40–70%	[73]
**Protein Kinases**					
JNK/PARP14 (Suppression)	Human liver Hep3B and Huh7 carcinomas	PYKM2	Protein content	Up ~1–1.4 times	[74]
			Activity	Up ~1.6 times	
mTOR	Human cervix HeLa carcinoma; human myeloid MOLM-14 leukemia	GLUT1, PFK1, Glc6PDH, R5PE	mRNA content	Up ~1–3 times	[75,76]
			Glucose consumption	Up ~2 times	

Hypoxia corresponds to 0.1–1% O_2_ over 24 h. Abbreviations: ALDO, aldolase; ECAR, extracellular acidification rate; ENO, enolase; Fru-2,6-BP2, fructose-2,6-bisphosphate; Fru2,6BPase, fructose 2,6 biphosphatase; Glc6PDH, glucose-6-phosphate dehydrogenase; GAPDH, glyceraldehyde 3 phosphate dehydrogenase; GLUT, glucose transporter; GS, glycogen synthase; HK, hexokinase; HPI, hexose-phosphate isomerase; LDH, lactate dehydrogenase; MCT, monocarboxylate transporter; PFK-L, phosphofructokinase liver isoform; PFK-P, phosphofructokinase platelet isoform; PGM, phosphoglucomutase; PGK, phosphoglycerate kinase; R5PE, ribulose-5-phosphate epimerase; PYK, pyruvate kinase (L/R/M: isozymes R and L or isoform M2); TPI, triosephosphate isomerase.

**Table 3:** Transcription regulators of cancer oxidative phosphorylation.

Transcription Regulator (Protein)	Cancer Cell	Target	Measured Parameter	Variation	Refs.
**Transcription Factors**					
HIF-1α (Hypoxia)	Human Burkitt’s P493-6 lymphoma	PDK	mRNA content	Up 4times	[80]
	Kidney RCC4, cervix HeLa, liver Hep3B, lung A495, colon HCT116 carcinomas	COX4-2	mRNA content	Up 2 times	[81]
	Human Burkitt’s P493-6 lymphoma, renal clear cell RCCA, kidney RCC4, cervix HeLa, liver Hep3B, lung A495, kidney RCC4, colon HCT116, breast T47D, MDA-MB-468 and MDA-MB-231 carcinomas, fibrosarcoma HT1080	BNIP3	mRNA content	Up 1–5 times	[81,82,83]
		BNIP3	Protein content	Up 0.5–3 times	
		COX4-1	Protein content	Down 80%	
		PDH	Activity	Down 50%	
			Total oxygen consumption	Down 80%	
p53 (Normoxia)	Nonmutant p53 human breast MCF-7, human colon HCT116 carcinomas	PDK2	Protein content	Down 75%	[84]
	Nonmutant p53 human liver HepG2; human colon HTC116 and H460, human cervix HeLa, human nonsmall-cell lung H1299, human large-cell lung H460 carcinomas	GA, SOC2c, ND1, COX4, 2OGDH, ATPS, AIF, Parkin	mRNA content	Up 3–12 times	[50,85,86,87,88]
			Protein content	Up two times	
			Total oxygen consumption	Up 0.5-fold	
			Δψm and OxPhos	Up 3 times	
			Glutaminolysis	Up 6 times	
p53 (Hypoxia)	Nonmutant cervix human carcinoma (HeLa)	COX4, 2OGDH and ATPS	Protein content	Down 40–90%	[52]
			Δψm and OxPhos flux	Down 75–85%	
p53 (Normoxia/hypoxia)	Mutant p53 ^R248Q^ human cervix HeLa carcinoma	2OGDH, GA, ND1, COX4, ATPS	Protein content	Down 10–50%	[52]
			Total oxygen consumption, Δψm and OxPhos flux	Down 50%	
PGC1-α	Human prostate PC3 carcinoma overexpressing PGC1α	SDH, IDH3, AAT	mRNA content	Up 5 times	[89]
		OAA, fum, mal, ATP	Metabolite content	Up 1–2 times	
			β-oxidation and OxPhos fluxes	Up 0.5–2.2-fold	
NRF-1	Human cervix HeLa cancer	COX, ND1; SDH; bc-1 complex; ATPS	Gene promoter activity *	Up 2–10 times	[90,91]
	Human breast MDA-MB-231 knockdown NRF-1 carcinoma	ATP	Metabolite content Total oxygen consumption	Down 40% 20%	[19]
TFAM (Knockdown)	Human liver Hep-2, human lung A549, human laryngeal HNE2 carcinomas		mtDNA content	Down 60%	[54,92,93]
			Total oxygen consumption	Down 40%	
STAT3	Human bladder T24 carcinoma	ND1, SDH	Activity OxPhos flux	Up 40–80% Up 70%	[94,95]
FOXO3a	Human colon DLD-1 carcinoma	Cyt *c*, ND1, FH	mRNA content Total oxygen consumption	Down 40% Down 75%	[96]
	Human colon DLD-1 carcinoma	PDK4	mRNA content	Up 1.5 times	
E2F	Human sarcoma Saos2	ND1, ACO, FH	mRNA content	Up 4 times	[97]
AR	Human prostate LNCaP and LAPC4 carcinoma	2OGDH, FH, ND1, ATPS	mRNA content OxPhos	Up > 2 times Up 2 times	[59,60]
ER	Human breast MCF7; human lung H1793 carcinoma; ERα-transformed MDA-MB-231	TNRF, COX	mRNA content	Up 0.8–5 times	[98,99,100]
			mtDNA content	Up 0.6-fold	
		HACoADH activity	Activity	Up 0.5 times	
			Total oxygen consumption	Up 0.8-fold	
PPARs	Human cervix HeLa; human osteosarcoma 143B; human breast MDA-MB-231 exposed to PPAR pan-agonist	CS, CPT-I and CPTII, COX	Activity	Up 0.1–1-fold	[101]
			Cellular ATP, Δψm	Up 10–40%	
**p53-Induced Phosphatase**					
TIGAR	Human glioma T98G and LNT-299 overexpressing TIGAR		Total oxygen consumption and OxPhos	Up 10–50%	[102]
**Oncogenes**					
c-MYC	Human Burkitt’s P493 lymphoma, glioma SF188, human-transformed CRL-2091 fibroblasts; rat TGR1-transformed fibroblasts	Mitochondrial biogenesis proteins; mitochondrial stability proteins; COX4, PDH, FH, ATPS, GA, glutamine transporters SLC38A5 and SLC1A5	mRNA content	Up 2–5 times	[103,104,105,106]
		GA	Activity	Up 2.5-fold	
		Acetyl-CoA	Metabolite content	Up 1.6-fold	
HRAS	No mutant HRAS-transformed mouse 3T3 fibroblasts	SDH, COX4	Protein content	Up 0.2–0.4-fold	[107,108,109]
			OxPhos flux	Up 0.5-fold	
	Mutant HRAS ^G12V^ and ^Q61L^ mouse 3T3 fibroblasts		OxPhos flux Oxygen consumption	Down 30–50%	
	Mutant HRAS ^G12V^-transformed human bronchial epithelial NHBE cells		OxPhos flux	Up 0.5-fold	[110]
KRAS	Nonmutant KRAS-transformed mouse 3T3 fibroblasts	ND1	Activity	Down 20%	[111]
			Oxygen consumption	Down 30%	
	Mutant KRAS ^G12V^-transformed human embryonic 239 kidney	ND1	Protein content	Down 25%	[112]
			Oxygen consumption	Down 60%	
**Tumor Suppressor**					
RB inactivation	Human breast MDA-MB-231 carcinoma	ATP	Metabolite content	Up 25 %	[113]
RB	Human osteosarcoma Saos2 and U2OS	BNIP3	Protein content	Down 50–70%	[114]
PTEN	Human glioma SF767, A172, and U87MG	ND1, SDH, bc1 complex, COX, ATPS	Protein content OxPhos flux	Up 30–80% Up 40%	[115,116]
**Protein Kinase**					
JNK	Human breast BT-549, MDA-MB-231 carcinoma	GA	mRNA content	Up 2 times	[117,118]
	Human cervix Hela carcinoma	ND1	Activity Oxygen consumption and OxPhos	Down 30% Down 20%	
mTOR	Human breast MCF7 carcinoma	ND1, IDH3, COX, ATPS	mRNA content	Up 0.6–2 times	[119,120]
	Human kidney HEK-293 carcinoma	ATG13	Protein content	Down 70%	[121]
**Plasma Membrane Receptors**					
Notch1 blocking	Human breast MDA-MB-231 carcinoma		Total oxygen consumption	Down 50%	[122]

Abbreviations: 2OGDH, 2 oxoglutarate dehydrogenase; ACO, aconitase; AIF, apoptosis-inducing factor; ATG13, autophagy-related protein 13; ATPS, adenosine triphosphate synthase; ATT, total aspartate aminotransferase; BNIP3, BCL2/adenovirus E1B 19 kDa protein-interacting protein 3; COX, cytochrome c oxidase; CPT-I and CPTII, carnitine palmitoyl transferases; CS, citrate synthase; FH, fumarate hydratase; fum, fumarate; GA, glutaminase; HACoADH, β-hydroxyacyl-CoA dehydrogenase; IDH, isocitrate dehydrogenase; mal, malate; ND1, NADH dehydrogenase; NRF-1, nuclear respiratory factor 1; OxPhos, oxidative phosphorylation; OAA, oxaloacetate; PDH, pyruvate dehydrogenase complex; PDK, pyruvate dehydrogenase kinase; SDH, succinate dehydrogenase; SLC38A5 and SLC1A5, Solute Carrier (Family 38 Member 5 and Family 1 Member 1); SOC2, Leucine-Rich Repeat Scaffold Protein. * Activities were normalized for transfection efficiency by Hirt DNA analysis [90].
